# A critical review of initial 3D printed products responding to COVID-19 health and supply chain challenges

**DOI:** 10.35241/emeraldopenres.13697.1

**Published:** 2020-05-13

**Authors:** James I. Novak, Jennifer Loy

**Affiliations:** 1 School of Engineering, Faculty of Science, Engineering and Built Environment, Deakin University, Geelong, VIC, 3216, Australia

**Keywords:** Additive Manufacturing, Coronavirus, Maker Movement, Online Collaboration, Open Source, Pandemic, Personal Protective Equipment (PPE), Product design

## Abstract

The COVID-19 pandemic significantly increased demand for medical and protective equipment by frontline health workers, as well as the general community, causing the supply chain to stretch beyond capacity, an issue further heightened by geographical and political lockdowns. Various 3D printing technologies were quickly utilised by businesses, institutions and individuals to manufacture a range of products on-demand, close to where they were needed. This study gathered data about 91 3D printed projects initiated prior to April 1, 2020, as the virus spread globally. It found that 60% of products were for personal protective equipment, of which 62% were 3D printed face shields. Fused filament fabrication was the most common 3D print technology used, and websites were the most popular means of centralising project information. The project data provides objective, quantitative insight balanced with qualitative critical review of the broad trends, opportunities and challenges that could be used by governments, health and medical bodies, manufacturing organisations and the 3D printing community to streamline the current response, as well as plan for future crises using a distributed, flexible manufacturing approach.

## Introduction

COVID-19 radically increased the demand for critical healthcare products at the start of 2020. The quantities of products required, and immediacy of the need, heightened by supply chain disruptions caused by practical and political barriers, forced communities to look elsewhere for short-term manufacturing solutions. The logistics of 3D printing (aka. additive manufacturing) identified it as a technology suited to such a crisis, providing products on-demand ( Quinlan *et al*., 2017; Thompson *et al*., 2016), in local proximity to that demand. No expensive tooling had to be pre-formed before production could begin. Individual parts could be 3D printed on the day requested and delivered where they were needed. The digital files describing products for 3D printing could be shared globally via the Internet and downloaded by others with a similar need, bypassing traditional supply chains through a distributed, collaborative and digitally-enabled manufacturing system.

However, the adoption of this strategy as a crisis response to production for critical healthcare products raises several concerns associated with regulation, ethics, intellectual property and accountability. These issues require objective evaluation to provide differentiation between a realistic provision of product, and good intentions that could potentially undermine the credibility of the technology for crisis manufacturing in the long term. Product development involves more than fabrication. Even pivoting production of a relatively simple part from one technology to another requires redesign specific to that technology. In conventional product development, healthcare products are subject to rigorous performance testing, both from a mechanical point of view, and for their particular use requirements within a clinical, or even domestic, environment. They are tightly regulated by governing bodies such as the Food and Drug Administration (FDA) in the United States, the Therapeutic Goods Administration (TGA) in Australia or the Medicines and Healthcare Products Regulatory Agency (MHRA) in the United Kingdom. Proving a product has met standards, particularly for a radically new design, or even a different manufacturing method, requires considerable time due to the range of scenarios that must be considered and validated. As a result of these processes, and the expertise necessary, this can be an expensive process.

Regulatory bodies like the FDA have increasingly recognised the emerging opportunities and challenges of 3D printed medical products ( Di Prima *et al*., 2016); however, these have largely been approached by established medical device manufacturers, and the introduction of 3D printing into medicine has been a cautious process spanning several decades. The ubiquity of desktop and personal 3D printers within the community, and manufacturers outside the medical device sector, has forced a rapid response from regulatory bodies as solutions to the challenges of COVID-19 preceded regulation. For example, the FDA established a Memorandum of Understanding with the Department of Veteran Affairs Innovation Ecosystem, National Institutes of Health (NIH) 3D Print Exchange and America Makes to provide guidelines and co-ordination on 3D printing of open-source medical products ( Food and Drug Administration, 2020). This was established on March 31, 2020, after COVID-19 had already been declared a pandemic weeks earlier on March 11 by the World Health Organisation (WHO), and 100,000 cases had been recorded worldwide on March 7 ( World Health Organisation, 2020). Similar responses also emerged in Australia ( Therapeutic Goods Administration, 2020) and Canada ( Government of Canada, 2020) with varying acceptance of 3D printed products to fill supply chain gaps during the COVID-19 crisis. One of the aims of this research was to track just how early 3D printed solutions emerged in relation to regulatory and WHO actions.

During a crisis situation, whether it be health, humanitarian or natural, the argument may be made that the need for assistive products outweighs concerns over regulation and the standard methods of product procurement. Research has already shown how small desktop 3D printers can be utilised in humanitarian crises ( Lipsky *et al*., 2019; Loy *et al*., 2016), providing short-term solutions until supply chains can be re-established, or more permanent solutions where aid may be unavailable or not of a safe standard. Products have included low-risk items, like connectors for water pipes ( Loy *et al*., 2016), as well as medical supplies like umbilical cord clamps and prosthetics ( Saripalle *et al*., 2016). However, 3D printing within such contexts has been limited to short, isolated case studies. COVID-19 has occurred on a global scale, with 3D printers and associated expertise already embedded within many Western communities, including homes, schools, universities, small and medium enterprises (SMEs), large-scale manufacturers and dedicated 3D printing bureaus. Alongside local expertise, online 3D printing communities have matured over the last decade to connect people and share files and knowledge ( Novak & Bardini, 2019; Özkil, 2017). Therefore, the COVID-19 situation is unlike anything that has come before.

Beyond the need to quantify the 3D printing response to COVID-19 during the first months of 2020 as the virus spread around the globe, this study also documented the types of products being 3D printed, and the 3D print technologies being utilised. During a time of crisis and emotion, an objective analysis that provides governments, health and medical bodies, manufacturing organisations and the 3D printing community with clarity on how 3D printing was utilised will help provide guidance for how regulatory bodies may modify their response to 3D printing of medical and health devices, as well as plan for future health, humanitarian or natural disasters that could be better responded to by an organised, proactive response leveraging 3D printing technology. Furthermore, the organisational tools for each 3D printing project were recorded in order to understand the principal means of collaboration that shaped the immediate 3D printing response. While challenging to document an unfolding crisis, the findings from this study will help inform the ongoing COVID-19 response and identify some of the benefits and shortcomings of 3D printing in the 2020 context. It also identified areas for continued research as the crisis continues, recommending a realistic 3D printing crisis response strategy, with product development, mobilisation and validation integrated into a product service system.

## Methods

In order to evaluate a real-time, immediate global challenge, correspondingly real-time resources were required to conduct this study. A mixed-methods quantitative and qualitative approach was taken to analyse the content of news media and social media, a well-established and popular approach to research of non-academic sources ( Graffigna & Riva, 2015; Snelson, 2016). The quantitative search for projects meeting inclusion criteria was conducted in two phases: The first search phase was broad and involved Google searches using the keywords *3D printing* with either *COVID-19* or *Coronavirus*, conducted by one of the authors during April 20–24, 2020. A date limitation of results appearing before April 1, 2020, was used to filter results, and the primary inclusion criteria at this stage was that the product had to be end-use (i.e. not just utilising 3D printing to prototype). The first ten pages of results were read by one of the authors to identify individuals, companies and projects that were utilising 3D printing to produce products to assist with the COVID-19 health crisis. In particular, 3D printing news websites such as 3Dprint.com and 3D Printing Industry, which appeared in the search, had already begun compiling regularly updated lists of projects, which provided a significant number of results for this study.

**Figure 1.**
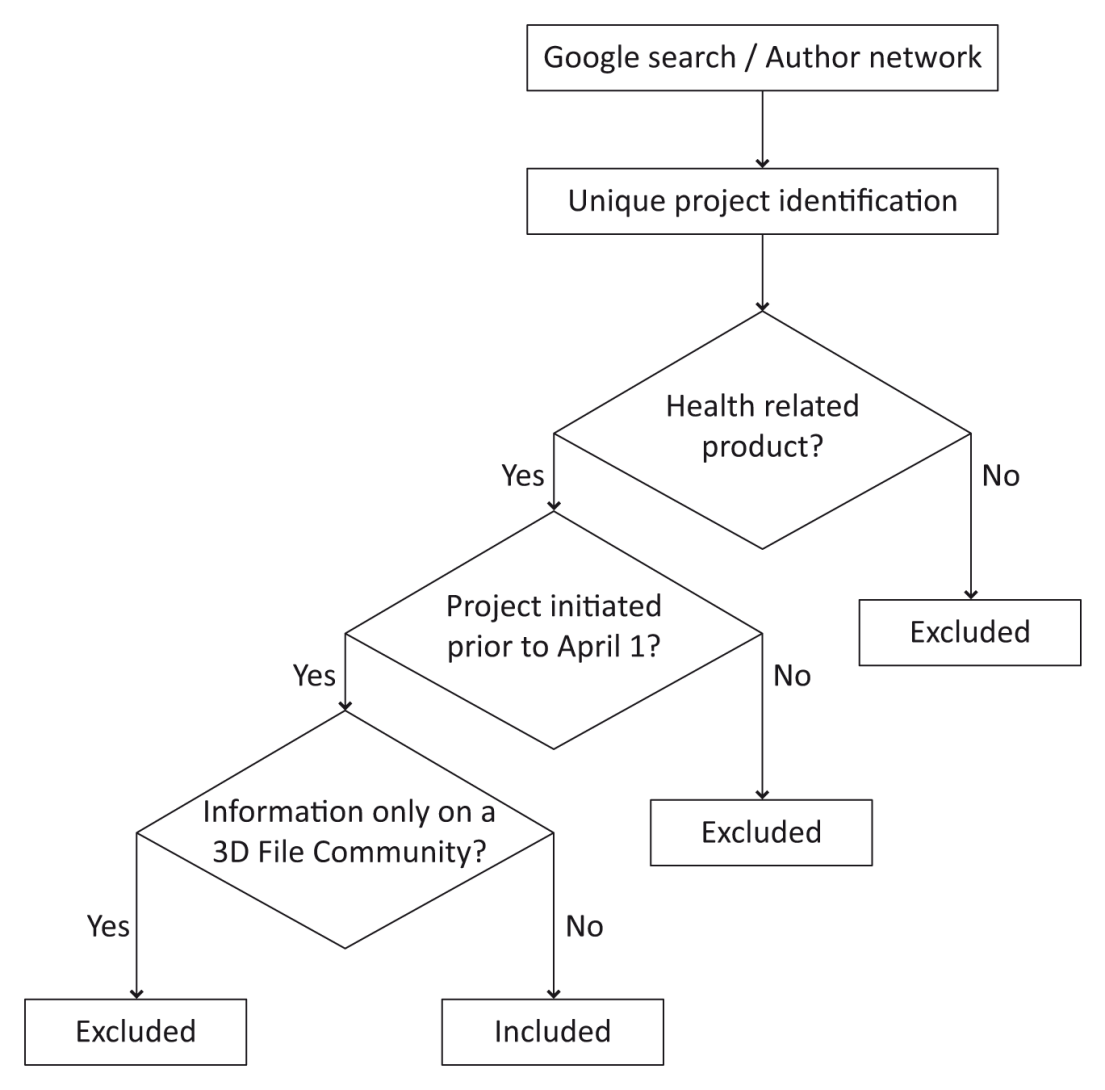
Flowchart for project inclusion strategy.

The second search phase involved both authors performing specific Google searches for companies appearing in the phase one results to find out details about individual responses to the pandemic that were published on company websites or social media, including Twitter and Facebook. Each author independently reviewed a selection of the companies identified, filtering results through the process shown in Figure 1. Any uncertainty was shared with the other author for clarification against the inclusion criteria, and agreement negotiated about the inclusion or exclusion status. The first filter was the requirement to be a health or medical product, as defined by the Therapeutic Goods Act 1989 ( Australian Government, 2019) in Section 41BD. The second filter was that the project had to have a record prior to April 1, 2020. This could be a blog post, news story, company announcement, or post on social media such as Twitter, Facebook, Instagram or LinkedIn. If it was unclear exactly when a project began, the earliest public record was taken as the start of the project, for example the date a news story was published, or a Tweet made. If the project could not be traced back to having origins prior to April 1, it was excluded from this study.

Projects passing these filters were then required to have documentation outside of a 3D printing file sharing community such as Thingiverse; for example, a news story, unique website or Git. While Thingiverse is the largest 3D printing file sharing community ( Alcock *et al.*, 2016; Novak & Bardini, 2019), files uploaded to the platform do not undergo public scrutiny or professional evaluation, and often provide little or no information about how to print or assemble the product, or even any evidence of the designer having 3D printed the product for themselves. In order to be included in this critical review, some level of qualitative evaluation, even if only by the news media or public on social media, was required. The only exclusion from this criterion were projects posted to NIH 3D Print Exchange, a biomedical 3D file sharing community that established a specific COVID-19 evaluation platform in collaboration with the Veterans Healthcare Administration to qualify designs as being suitable for “Clinical Use” or “Community Use” ( NIH 3D Print Exchange, 2020). Projects that were copies of established products, or that did not clarify how they were different from an existing product (known as a remix, fork or mashup ( Novak, 2019; Oehlberg *et al.*, 2015)), were also not included. These copies were identified against the data collected by the authors in the results as they traced projects to their source and the original maker or company who created a project.

Projects passing these filters were then compiled from both authors to create a master list, documented chronologically in Microsoft Excel (Microsoft Office 365 version) by the date they began, or were first made public. Key information about the type of product, print technology and primary method of organisation or collaboration was then documented in full by the principal author.

## Results

**Figure 2.**
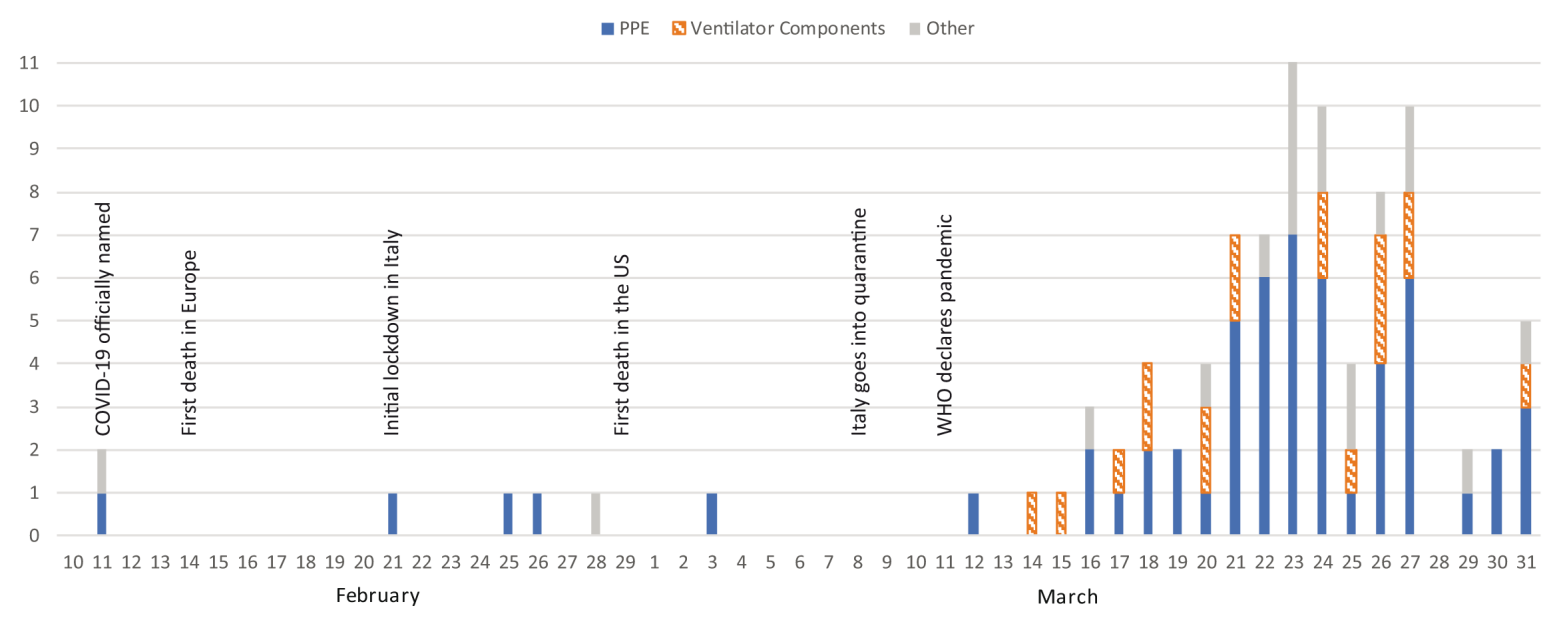
COVID-19 3D printing projects mapped against date of release/publication PPE, personal protective equipment.

In total, 91 unique 3D printing projects met inclusion criteria, documented in full as *Underlying data* ( Novak & Loy, 2020), and were summarised in the Figure 2 timeline. February 11 was the first recorded evidence of 3D printing being used to solve COVID-19 related challenges, with goggles for healthcare workers being produced by Hunan Vanguard Group Co., Ltd in China ( Creality, 2020), and 3D printed concrete isolation houses for Xianning Central Hospital in Hubei, China ( Winsun, 2020). It was not until the World Health Organisation (WHO) pandemic declaration on March 11 that growth in the number of projects was observed, with 92% of the documented projects occurring after this date. Of the seven projects occurring before the WHO declaration, only one was based in a company outside of Asia. In total, 60% (n=55) of projects were for PPE products, 20% (n=18) were for ventilator components and 20% (n=18) were for a range of other products, including nasopharyngeal (nasal) swabs and hands-free door openers.

**Figure 3.**
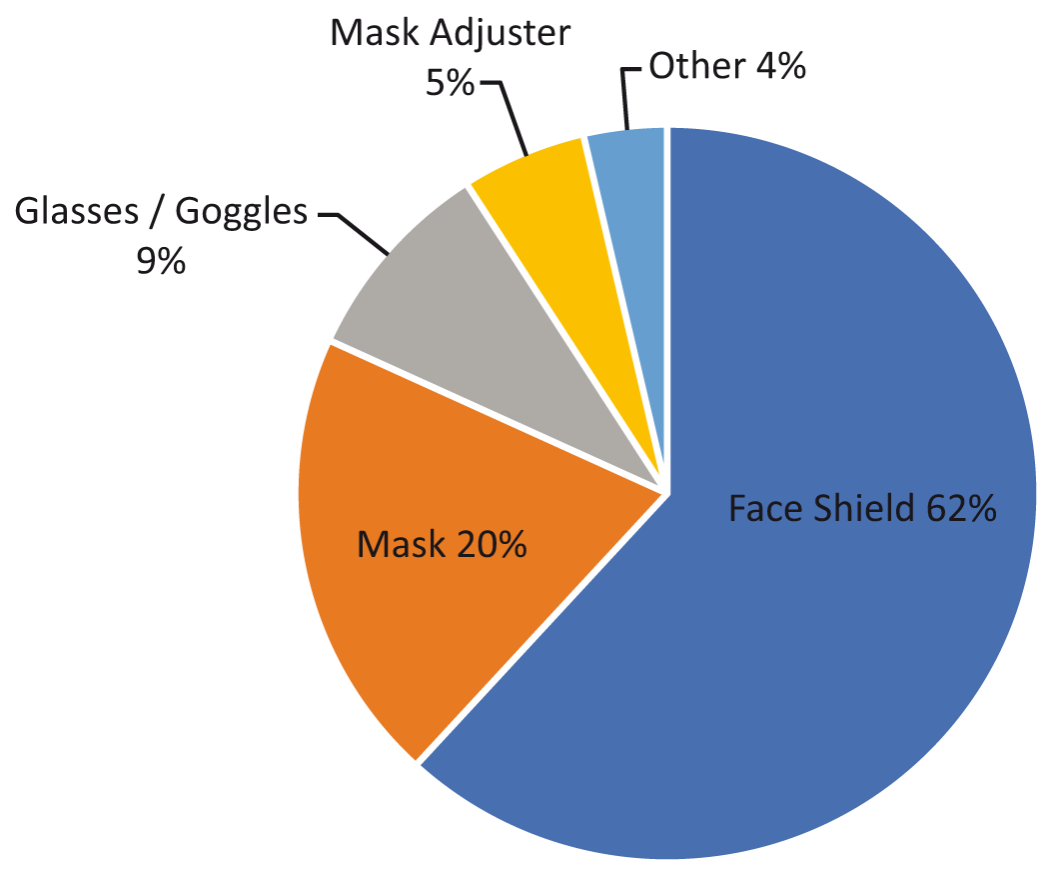
Types of PPE products being 3D printed during February-March 2020. PPE, personal protective equipment.

Figure 3 provides a more detailed analysis of the PPE products identified in this review, with the majority of projects being for face shields (62%, n=34). 3D printing is typically used to produce the head-worn frame of a face shield, with a clear plastic sheet material fixed to the frame to protect the wearer’s face from airborne material that may be contaminated with the COVID-19 virus. Face shields accounted for 37% of total projects in this review with the first account of a 3D printed face shield being on February 25 from The Hong Kong Polytechnic University ( Cheng, 2020).

**Figure 4.**
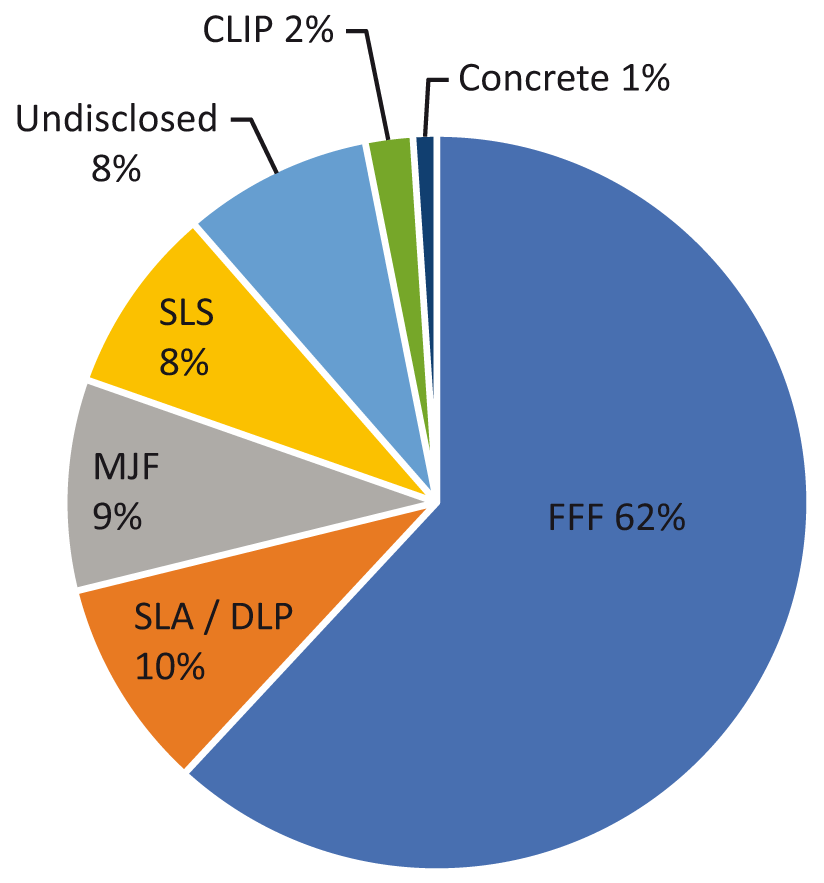
3D printing technologies utilised in projects. FFF, fused filament fabrication; SLA, stereolithography; DLP, digital light projection; MJF, multi jet fusion; SLS, selective laser sintering; CLIP, continuous liquid interface production.

From a technical perspective, Figure 4 provides a breakdown of the 3D printing technologies being utilised for each project. It is important to note that several projects included several variations of a product designed for different 3D print technologies, for example the Materialise hands-free door opener which had fused filament fabrication (FFF), selective laser sintering (SLS) and multi jet fusion (MJF) versions ( Materialise, 2020). This resulted in 97 records for the 91 projects. While a single 3D design may be printed through a range of different processes, this study only recorded the technology specified or indicated by the main project documentation. If categorised more generally in line with ISO/ASTM 52900 standards and the seven 3D printing process categories, three categories are represented in the projects in this review: material extrusion (FFF and concrete = 63%), powder bed fusion (MJF and SLS = 17%) and vat photopolymerisation (stereolithography [SLA] / digital light projection [DLP] and continuous liquid interface production [CLIP] = 12%). 8% of projects provided no specific detail about the 3D printing technology utilised or required to produce them.

The most popular product to 3D print using FFF was face shields (52%, n=31), while for SLA/DLP it was components for ventilators (44%, n=4). The low number of SLS products were spread across several categories, although hands-free door openers were the most popular (38%, n=3), while MJF had a more even distribution of products with ventilator components, mask components and face shields each representing 22% (n=2). The two CLIP projects were nasopharyngeal swabs and face shields.

**Figure 5.**
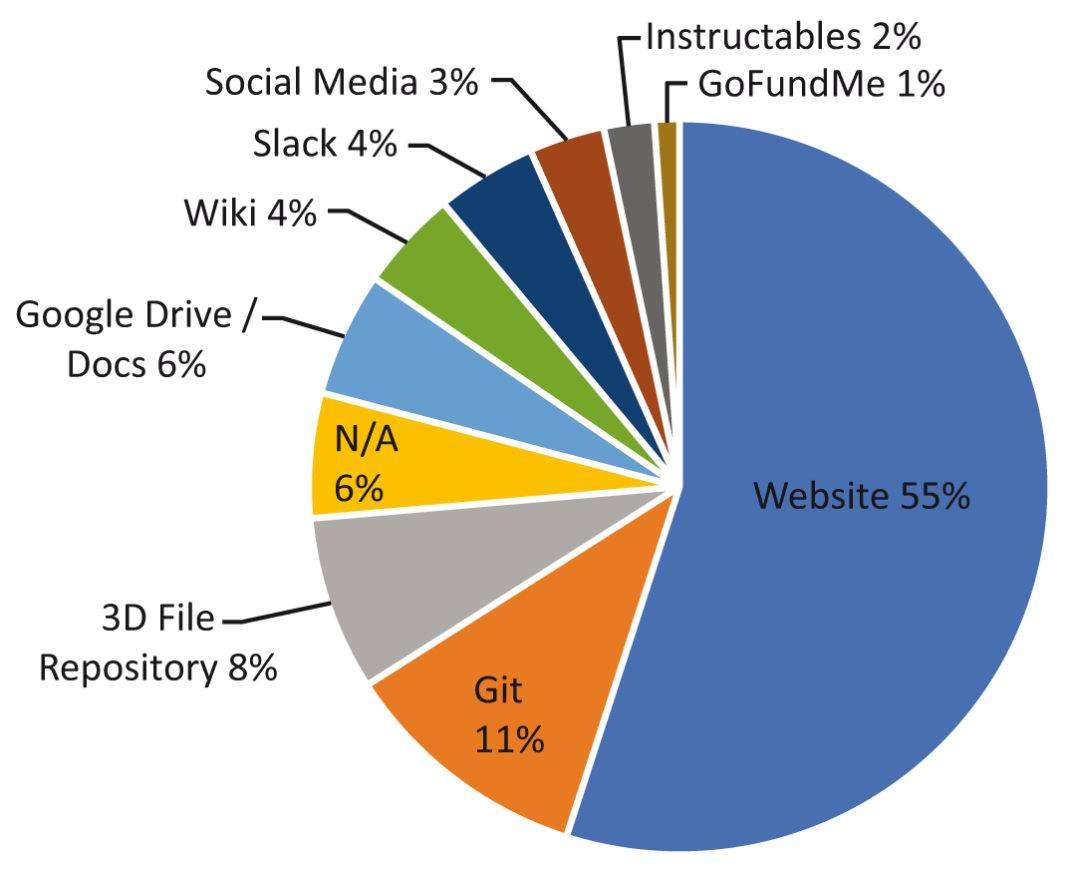
Principal organisation/collaboration tool for each project.

From an organisational perspective, Figure 5 shows the principal tool used to manage each project, bring collaborators together, or report results. While most projects leveraged multiple platforms in order to extend awareness and involvement, Figure 5 shows that a dedicated website was the most popular means of centralising and reporting information, accounting for 55% (n=50) of projects. In some cases, this was the creation of a new website specifically for the project being developed, and in other cases it was an added page on an established company website. GitHub and GitLab were the principle organisational tools used in 11% (n=10) of projects, while 3D file repositories were the third most popular means of collaboration with 8% (n=7) of projects, predominantly NIH 3D Print Exchange. Many other projects used 3D file repositories such as Prusa Printers or Thingiverse as secondary means of collaboration and communication.

6% of projects did not have a clear central organisation mechanism, and this figure aligns with the 8% of undisclosed 3D print technologies from Figure 4, typically reliant upon media reporting that lacked technical detail. This was more likely in projects documented before the WHO declaration, with four of the seven projects (57%) from this period only found to be reported through online news media.

## Discussion

Given the severe and global nature of COVID-19, and the altruistic desire of individuals and companies to assist their community in any way possible, it was no surprise that those with 3D design and printing expertise utilised their skills to address deficits in the product supply chain during the first months of 2020. In the US alone, estimates suggest there are 47,000 industrial 3D printers that may be largely idle as companies reduce their manufacturing capacity during the pandemic ( Feldman, 2020), most of which could be deployed to manufacture medical products in short supply. Many of these companies and institutions featured in this review, and at the time of writing, many more joined the call during April to shift their operations towards producing medical equipment. However, this study revealed that it was not just industrial 3D printing being used to produce products, but a significant quantity of “makers” ( Anderson, 2012; Dougherty, 2016) and hobbyists with one or several desktop 3D printers who were contributing to the COVID-19 response. This was reflected in the prominence of FFF technology in the majority of projects in this review, with FFF being the most common form of 3D printing due to the relatively cheap and simple hardware mechanisms, and the expiry of key patents over a decade ago ( Gibson *et al.*, 2015; Novak & O'Neill, 2019) that saw a growth in competition and variety of machines on the market.

Given the breadth of FFF 3D printing technologies, ranging from small desktop machines costing hundreds of dollars, to robotic arms and large industrial machines costing tens of thousands of dollars, a side-effect within the COVID-19 context is the broad variety of different designs addressing the same problem, for example the 34 different face shield projects recorded in this review. While some designs, such as the popular Prusa RC3 face shield ( Prusa Research, 2020) and IC3D Budmen face shield ( IC3D Industries, 2020), were the result of simultaneous invention, both dated to March 16, other variations to designs were a necessity in order to allow 3D printing on different machines with different capabilities. For example, the face shield by Nagami Design (2020) was a remix of the Prusa RC3, modified in order to be printable using a large robotic arm 3D printer rather than a small desktop machine. This may lead to some confusion for healthcare professionals and others less experienced with 3D printing, making the choice of design for their 3D printing capabilities, as well as their functional requirements, difficult. Some have also questioned the motivations of companies joining the 3D printing challenge for face shields or other equipment, suggesting that it may have become a marketing exercise that adds further confusion for those wanting to help 3D print supplies ( Peels, 2020; Ślusarczyk, 2020). The appearance of branding on many designs was evidence of this.

The overview data presented in Figure 2 shows several trends worth examination: Firstly, as mentioned previously, six out of the seven projects documented prior to the WHO declaration were based in Asia. This aligns with the geographical spread of COVID-19, reportedly stemming from Wuhan, China ( Sohrabi *et al*., 2020), and the local community response to challenges in accessing PPE and other medical products. As the virus spread globally, so too did the 3D printing response. The low figures throughout February and early March may indicate a lack of global urgency until more countries outside of Asia were affected by COVID-19, with many governments criticised for reacting slowly to the virus ( Cowper, 2020). However, they may also be a symptom of the method of this study, specifically, the use of Western search tools like Google and Twitter that may have restrictions in countries like China, as well as the use of the English language, which may not identify news and projects written in another language. Further research is needed to accurately understand how 3D printing may have been used in countries like China.

Another trend from Figure 2 is the peak of project reporting on March 23, before a sharp decline in the last days of March. Several factors may account for this, including the fact that once projects were established, there was less need for a company or individual to create a new solution, instead joining an existing project that had already gained momentum. The decline in late March also aligned with regulation agencies such as the FDA releasing their 3D printing guidelines for medical equipment ( Food and Drug Administration, 2020), providing information that may have caused some manufacturers, designers and makers to reconsider their projects. This could be found upon review of some projects in this study, for example the EnvisionTEC nasopharyngeal swabs could not be downloaded without proof of having an FDA registered account to produce medical devices, while their face shield required anyone downloading the design to acknowledge and agree to the terms outlined in a release waiver ( EnvisionTEC, 2020). The partnership between the NIH 3D Print Exchange, FDA, America Makes and Veterans Affairs was announced on March 31, and as of April 29, only 16 projects had passed clinical review and five had been cleared for community use. Neither of these categories are comparable to FDA certification. Numerous projects have sought quasi-validation through endorsement from medical practitioners and approval from hospitals or health agencies, neither of which are regulated.

In terms of the specific products being 3D printed from this review, the dominance of PPE is a reflection of the relative simplicity of designs like face shields and goggles compared to more complex medical equipment like ventilators, which may combine 3D printed elements with electronics and other manufacturing technologies, or require the greater precision of parts printed with industrial 3D printers. It is also a reflection of the lower-risk classifications attributed to PPE compared to ventilators within FDA and other regulatory frameworks. A survey of the Make: community on April 7 ( Kraft, 2020) also found a large number of people focusing their efforts on PPE, with 96% of the products being worked on PPE, compared to 60% in this study. However, the percentage of PPE products that were face shields were similar, with 61% recorded by the Make: community, compared with 62% in this review, providing some validation to this study. The difference between overall project categories may be due to the nature of the Make: community, which is largely made up of individuals with desktop technologies like FFF, whereas this review considered large manufacturers and institutions who were also developing more complex projects. The data from the Make: community also considered all forms of production, not just 3D printing.

One of the challenges with this study was the reliance on fluid mediums like websites, Tweets, blog posts and Facebook groups in order to track projects, and define when they began. It is likely that many projects began earlier than reported, only shared online once the creator was happy with the outcome. However, given the rapid pace of developments through this pandemic, and regular reports of 3D printed projects being developed in a matter of days, it is unlikely that minor variations to the launch date of some projects would affect the overall trends or results from those reported. These mediums also made identification of the principle organisation platform challenging in some cases, with many projects utilising a broad range of online media that changed as projects matured, or were developed collaboratively by several companies without a clear lead, or with partnerships that formed after the initial project was launched. While every effort was made to accurately classify projects in this review, it is possible that some information was missed, and indeed, will change by the time this research is published.

While this review provides an overview of the broad trends related to the 3D printing of health and medical products during the first months of the COVID-19 pandemic, ongoing research is needed to continue monitoring 3D printed products throughout the pandemic to understand longitudinal trends. For example, does the initial hype from March subside and a more stable pattern of research and collaboration continue through April and the following months? Do projects consolidate and merge, with others ending as regulations tighten, or traditional supply chains stabilise? It will also be necessary to analyse 3D printed products and validate them, particularly as the health crisis continues for months or even years. Initial 3D printing projects, while well intentioned, were largely unregulated and a reflexive response to direct and immediate needs. As supplies stabilise, and the infection curve flattens, more time and resources can be devoted to research, building upon the NIH 3D Print Exchange database of approved designs, perhaps developing an approved FDA or TGA database of designs as well as 3D print technologies and materials. These may be necessary for any future outbreaks of the virus, as well as allowing for better preparation for future health, humanitarian and natural disaster crises that may require a similarly rapid response to equipment shortages.

Elements of manufacturing in a post-COVID-19 future may look very different to pre-COVID-19. 3D printing could be central to new ways of thinking about making and distribution, but only if it is successful in avoiding being undermined by hype. Researchers, manufacturers and those with a vested interest in 3D printing must commit to building products designed for each additive manufacturing technology, with systems that maximise potential, whilst shouldering the responsibilities involved in producing viable, qualified products that can be relied on by society whether in a crisis or not.

## Conclusion

3D printing provides a novel and distributed means of producing health and medical equipment, especially when established supply chains are under distress, and supply cannot keep up with demand. The 3D printing response to COVID-19 challenges during the first months of 2020 provides an opportunity for the review of design for 3D printing, how it is being used in a distributed manufacturing, healthcare context, as well as how it is perceived. Data from this study categorised 91 3D printing projects that were initiated prior to April 1, finding that the most common type of product being produced was personal protective equipment, with face shields the most popular product overall. While the technology has matured significantly over the last decade, FFF remains the most common technology being used to produce COVID-19 products, arguably indicative of the level of sophistication of the products determined suitable for 3D printing at this time. It is also indicative of the need for further workforce development in the adoption of different forms of 3D printing technology overall. For 3D printing to become a viable, credible alternative in emergency response conditions, there needs to be a significant investment in the development of 3D printed products and a production and regulatory framework to support a design response in anticipation of calls for localised manufacturing during the next crisis.

## Data availability

### Underlying data

Figshare: Underlying Data: A critical review of initial 3D printed products responding to COVID-19 health and supply chain challenges. https://doi.org/10.6084/m9.figshare.12250913.v1 ( Novak & Loy, 2020)

This project contains the following underlying data:

- Underlying Data - 3D Print COVID-19.csv

Data are available under the terms of the Creative Commons Attribution 4.0 International license (CC-BY 4.0).
