# Supplementary material for: A critical review of initial 3D printed products responding to COVID-19 health and supply chain challenges
Source: Emerald Open Res. 2020 May 13;2:24. doi: 10.35241/emeraldopenres.13697.1 (PMC7292530; doi:10.35241/emeraldopenres.13697.1)
Supplement: Peer review history

# Peer review: Reviewer response for version 1

**DOI:** 10.21956/emeraldopenres.14771.r26802

**Published:** 2020-06-03

**Authors:** Lionel Dean

**Affiliations:** 1 School of Design, De Montfort University, Leicester, UK

This is a well-researched and documented study. Additive Manufacture/3D Printing has long been heralded as a disruptive technology than can bypass supply chains and respond to local need. The Covid-19 crisis provides an ideal case study and this report provides a balanced insightful assessment. It will hopefully lead to further longer-term study of impact.

Is the work clearly and accurately presented and does it cite the current literature?

Yes

If applicable, is the statistical analysis and its interpretation appropriate?

Yes

Are all the source data underlying the results available to ensure full reproducibility?

Yes

Is the study design appropriate and is the work technically sound?

Yes

Are the conclusions drawn adequately supported by the results?

Yes

Are sufficient details of methods and analysis provided to allow replication by others?

Yes

Reviewer Expertise:

Additive manufacture and the creative use of digital design and manufacturing technologies

I confirm that I have read this submission and believe that I have an appropriate level of expertise to confirm that it is of an acceptable scientific standard.

# Peer review: Reviewer response for version 1

**DOI:** 10.21956/emeraldopenres.14771.r26801

**Published:** 2020-05-27

**Authors:** Olaf Diegel

**Affiliations:** 1 University of Auckland, Auckland, New Zealand; 2 Lund University, Lund, Sweden

The paper is well written and easy to read. It presents a fairly comprehensive view of how 3D printing has been able to contribute towards helping with the COVID19 crisis. It also gives some good statistics and charts on the different areas and 3D printing technologies that have been used during the crisis.

 It would have been great to include some pictures of examples of COVID PPE and other equipment to help visualize the context, and to help highlight the differences between the results produced by some of the 3D printing technologies used. It would also have been good to see some mention of the challenges of sanitizing 3D printed PPE, as this is a greater or lesser issue depending on which technology is used and on whether the PPE is classified as disposable or reusable.

 Despite these comments, the paper makes a useful contribution to the field and is well worth the read.

Is the work clearly and accurately presented and does it cite the current literature?

Yes

If applicable, is the statistical analysis and its interpretation appropriate?

Yes

Are all the source data underlying the results available to ensure full reproducibility?

Yes

Is the study design appropriate and is the work technically sound?

Yes

Are the conclusions drawn adequately supported by the results?

Yes

Are sufficient details of methods and analysis provided to allow replication by others?

Yes

Reviewer Expertise:

Additive manufacturing, 3D printing , Rapid product development

I confirm that I have read this submission and believe that I have an appropriate level of expertise to confirm that it is of an acceptable scientific standard.

# Peer review: Reviewer response for version 1

**DOI:** 10.21956/emeraldopenres.14771.r26803

**Published:** 2020-05-14

**Authors:** Ismail Fidan

**Affiliations:** 1 Department of Manufacturing and Engineering Technology, Tennessee Technological University, Cookeville, TN, USA

It will be great if the article adds at least a figure showing a few examples of 3D printed products with their labeling.

 From our practices it was impossible to make the parts 100% 3D printed. We added externally purchased gaskets to our 3D printed masks and elastic bands to face shields. Lately, it was tough to find elastic bands, then, our solution was to redesign the headband so that it could function without any elastic band. That could be great if the authors can touchbase these points.

 There is a safety aspect of the developed products. Authors should briefly talk about the health and safety issues originated by the filaments used and their materials.

 Forbes published a very strong and impressive article about this topic. That is one of the highly read and cited articles. It is suggested authors use and cite some portions of it. It is at https://www.forbes.com/sites/tjmccue/2020/03/24/calling-all-makers-with-3d-printers-join-critical-mission-to-make-face-masks-and-shields-for-2020-healthcare-workers/#1bb025517500.

 The efforts of America Makes should be debriefed shortly. America Makes joined forces with the FDA, NIH, and VA to be the bridge connecting the need for medical PPE with manufacturers capable of 3D printing safe PPE for medical providers.

Is the work clearly and accurately presented and does it cite the current literature?

Yes

If applicable, is the statistical analysis and its interpretation appropriate?

Yes

Are all the source data underlying the results available to ensure full reproducibility?

Yes

Is the study design appropriate and is the work technically sound?

Yes

Are the conclusions drawn adequately supported by the results?

Yes

Are sufficient details of methods and analysis provided to allow replication by others?

Yes

Reviewer Expertise:

3D Printing

I confirm that I have read this submission and believe that I have an appropriate level of expertise to confirm that it is of an acceptable scientific standard.
